# Cardiopulmonary exercise testing excludes significant disease in patients recovering from COVID-19

**DOI:** 10.1136/military-2022-002193

**Published:** 2022-11-28

**Authors:** D A Holdsworth, R M Barker-Davies, R R Chamley, O O’Sullivan, P Ladlow, S May, A D Houston, J Mulae, C Xie, M Cranley, E Sellon, J Naylor, M Halle, G Parati, C Davos, O J Rider, A B Bennett, E D Nicol

**Affiliations:** 1 Oxford University Hospitals NHS Foundation Trust, Oxford, UK; 2 Royal Centre for Defence Medicine, Birmingham, UK; 3 Academic Department of Military Rehabilitation, Defence Medical Rehabilitation Centre, Loughborough, UK; 4 Oxford Centre for Clincal Magnetic Resonance Research, University of Oxford, Oxford, UK; 5 Defence Medical Rehabilitation Centre Stanford Hall, Loughborough, UK; 6 Academic Department of Military Rehabilitation, Defence Medical Services, Loughborough, UK; 7 Klinikum rechts der Isar der Technischen Universität München, Munchen, Germany; 8 Università degli Studi di Milano-Bicocca, Milano, Italy; 9 Academy of Athens Biomedical Research Foundation, Athens, Greece; 10 School of Biomedical Engineering and Imaging Sciences, King's College, London, UK

**Keywords:** COVID-19, Respiratory physiology, rehabilitation medicine, adult cardiology

## Abstract

**Objective:**

Post-COVID-19 syndrome presents a health and economic challenge affecting ~10% of patients recovering from COVID-19. Accurate assessment of patients with post-COVID-19 syndrome is complicated by health anxiety and coincident symptomatic autonomic dysfunction. We sought to determine whether either symptoms or objective cardiopulmonary exercise testing could predict clinically significant findings.

**Methods:**

113 consecutive military patients were assessed in a comprehensive clinical pathway. This included symptom reporting, history, examination, spirometry, echocardiography and cardiopulmonary exercise testing (CPET) in all, with chest CT, dual-energy CT pulmonary angiography and cardiac MRI where indicated. Symptoms, CPET findings and presence/absence of significant pathology were reviewed. Data were analysed to identify diagnostic strategies that may be used to exclude significant disease.

**Results:**

7/113 (6%) patients had clinically significant disease adjudicated by cardiothoracic multidisciplinary team (MDT). These patients had reduced fitness (V̇O_2_ 26.7 (±5.1) vs 34.6 (±7.0) mL/kg/min; p=0.002) and functional capacity (peak power 200 (±36) vs 247 (±55) W; p=0.026) compared with those without significant disease. Simple CPET criteria (oxygen uptake (V̇O_2_) >100% predicted and minute ventilation (VE)/carbon dioxide elimination (V̇CO_2_) slope <30.0 or VE/V̇CO_2_ slope <35.0 in isolation) excluded significant disease with sensitivity and specificity of 86% and 83%, respectively (area under the receiver operating characteristic curve (AUC) 0.89). The addition of capillary blood gases to estimate alveolar–arterial gradient improved diagnostic performance to 100% sensitivity and 78% specificity (AUC 0.92). Symptoms and spirometry did not discriminate significant disease.

**Conclusions:**

In a population recovering from SARS-CoV-2, there is reassuringly little organ pathology. CPET and functional capacity testing, but not reported symptoms, permit the exclusion of clinically significant disease.

**WHAT IS ALREADY KNOWN ON THIS TOPIC**

- Persistent, life-limiting symptoms are common following COVID-19.
- The combination of organ pathology and postviral fatigue makes it difficult to confidently establish the cause of breathlessness, chest pain, palpitations and fatigue.

**WHAT THIS STUDY ADDS**

- Cardiopulmonary exercise testing (CPET) parameters can sensitively exclude significant lung and heart pathology in a working age cohort of patients recovering from COVID-19.

**HOW THIS STUDY MIGHT AFFECT RESEARCH, PRACTICE OR POLICY**

- CPET is a safe, cheap, widely accessible and objective test, which can exclude significant lung and heart pathology post-COVID-19.
- CPET therefore also has the potential to identify the minority of individuals with significant cardiopulmonary disease and reassure the remaining majority.

## Introduction

The COVID-19 pandemic has caused more than 600 million infections globally.^1^ Approximately 10% of infected individuals have protracted symptoms that persist beyond 12 weeks.^2–4^

SARS-CoV-2 is associated with multisystem internal organ involvement. From early in the pandemic, pathology has been described in the lung, heart, kidney and brain.^5–7^

In July 2020, the UK Armed Forces commissioned a pathway to exclude clinically significant internal organ pathology (clinically significant disease) for all personnel following a severe acute COVID-19 illness or with prolonged symptoms. These personnel were made ‘unfit for strenuous physical exertion’ and ‘medically non-deployable’ pending assessment.^8^

This pathway is deliberately centred on exercise testing. Strenuous physical activity is a core component of military service. Service personnel may be required to undertake maximal exercise in dangerous, remote environments, with limited local medical support. Cardiopulmonary exercise testing (CPET) is the gold-standard measure of maximal cardiorespiratory capacity.^9 10^ Furthermore, where capacity is limited, CPET can identify the physiological component responsible for that limitation (circulatory, ventilatory, peripheral) and in most cases will substantially narrow the differential diagnosis. This has proven value in the assessment of unexplained breathlessness^9 11^ and has been proposed for the evaluation of pulmonary function in patients recovering from COVID-19.^12^ CPET can also reveal disordered breathing (eg, inappropriate hyperventilation), which has been suggested to account for a significant proportion of findings in a group living with fatigue and anxiety.^13^

While persistent symptoms may be similar, a key challenge faced by clinicians managing patients following COVID-19 is the need to discriminate symptoms that are caused by physical organ damage, such as lung fibrosis and myocarditis, from those caused by postviral fatigue,^14^ which have different treatment pathways. This diagnostic dilemma is exacerbated by coexisting health anxiety, relating to concerns regarding employment, health and well-being.^15^ It is further complicated by the association of SARS-CoV-2 with autonomic dysregulation. Such ‘dysregulation’ is evidenced by the finding of abnormal heart rate responses to exercise in a high proportion of post-COVID patients in the absence of demonstrable structural pathology in the heart and lungs.^16^

Clinical testing of exercise capacity is an objective, cheap, reproducible, safe and prognostically powerful means to stratify clinical risk,^17 18^ especially in high-hazard occupations.^19^ CPET and invasive CPET have been used to investigate the nature of exertional limitation in post-COVID cohorts.^20–23^ The two small CPET studies (10 and 18 patients, respectively), which measured cardiac output concluded that exertional limitation results from impaired peripheral oxygen uptake.^20 21^ To date, no study has reported the diagnostic potential of post-COVID CPET findings in the prediction of persistent cardiopulmonary abnormalities. We hypothesised that CPET would be able to predict clinically significant lung and heart disease in patients recovering from COVID-19.

## Methods

### Patients

Patients with confirmed or probable COVID-19 were referred from primary care, in accordance with UK Defence policy.^24^ Policy directs the assessment of patients:

- Hospitalised with COVID-19 with a supplemental oxygen requirement.
- With community infection experiencing life-limiting symptoms >12 weeks after acute illness.
- Desaturating to ≤95% on peripheral pulse oximetry following 1 min of the Harvard step test.
- With chest pain and troponin rise or ECG changes during acute illness.

Severe acute illness was pragmatically defined by the need for an inpatient stay and the requirement for supplemental oxygen.

### Investigations and clinical review

Details of the 3-day residential assessment pathway have been described previously.^8^ Initial assessment includes standard observations, 12-lead ECG, transthoracic echocardiogram, 6-min walk test, routine blood tests, spirometry and CPET. Each patient has a consultation with both a consultant physician and a consultant in rehabilitation medicine. Patients are then discussed by the multidisciplinary team (MDT)—including chest physicians, cardiologists, rehabilitation consultants, radiologists and nurses. The team directs either discharge from the clinic, with a diagnosis and plan, or further investigations including CT thorax, dual-energy CT pulmonary angiography (CTPA) and cardiac MRI (CMR). These investigations take place the same week in an National Health Service (NHS) teaching hospital. The clinical process is completed by MDT discussion of all investigation findings the following week, resulting in a clinical diagnosis, clinical plan and recommended rehabilitation. Many clinical patients have also volunteered for a research study to determine the effects of COVID-19 on the service population. The occupational post-COVID-19 (M-COVID) study received favourable ethical opinion (1061/MODREC/20). It includes CT chest, CTPA and CMR for all patients.

### Patient symptoms

All patients completed standardised questionnaires to assess subjective breathlessness (modified BORG, 0–10 Breathlessness Scale), fatigue (Fatigue Assessment Scale),^25 26^ anxiety (generalised anxiety disorder assessment, GAD-7),^27^ depression (patient health questionnaire, PHQ-9)^28^ and post-traumatic stress (Posttraumatic stress disorder checklist for DSM-5, PCL-5).^29^

### Spirometry

Spirometry was performed, including contemporaneous height and weight measurement, using a Microlab ML 3500 spirometer (Microlab Europe, Baar, Switzerland) with repetition (minimum of three efforts) to achieve repeatable measurements in accordance with British Thoracic Society guidelines.

### Transthoracic echocardiogram

Echocardiograms were acquired with a Philips EPIQ 5 ultrasound scanner (Philips, Amsterdam, The Netherlands) by accredited echocardiographers working to the standard British Society of Echocardiography transthoracic echocardiogram dataset (2013).

### Six-min walk test

The 6-min walk test was conducted in accordance with American Thoracic Society guidelines (2002), including prescribed standardised instruction text.

### Cardiopulmonary exercise testing

Oxygen uptake (V̇O_2_) peak, peak workload achieved, ventilatory efficiency, breathing reserve and oxygen pulse (V̇O_2_ peak/peak heart rate—which is correlated with cardiac stroke volume) were determined in a ramp-exercise protocol to volitional fatigue on an electromagnetically braked upright cycle ergometer (Lode Corival; Lode BV, Groningen, The Netherlands). The test commenced with a 2-min resting recording, followed by 2 min of unloaded pedalling, then a progressive ramp protocol (starting at 25 W with a ramp of 15–35 W/min) to achieve a test lasting 8–12 min. Measurements of breath composition, tidal volume and breathing frequency were performed by indirect calorimetry (Metalyzer 3B Cortex Biophysik, Leipzig, Germany). Maximal testing was defined as a peak respiratory exchange ratio (RER) ≥1.10 and/or plateau in oxygen uptake despite increasing workload. Predicted peak V̇O_2_, predicted peak workload and predicted O_2_ pulse (V̇O_2_/heart rate) are based on the Wasserman weight algorithm.^30^

### Cardiothoracic imaging

The chest CT examination comprised high-resolution CT (HRCT) and/or dual-energy CTPA (DECTPA) acquired using a dual-source CT (Siemens SOMATOM Drive; Siemens Healthineers, Erlangen, Germany). The interspaced HRCT protocol consisted of inspiratory 1 mm sections with 10 mm gap, followed by expiratory 1 mm section with 30 mm gap. Subsequent DECTPA (1 mm reconstructed slice thickness) and perfusion map were analysed using a dedicated workstation with Siemens Syngo.CT DE Lung Analysis postprocessing software. Cardiovascular MRI was performed on a Siemens 3T Trio scanner (Siemens Healthineers, Erlangen, Germany). In addition to standard cine imaging and function imaging, late gadolinium enhancement imaging was undertaken for cardiac fibrosis/scarring.

### Clinical adjudication of significant disease

Clinically significant disease was decided on the basis of history, clinical examination, initial investigations and the outcome of cross-sectional CT thorax and CMR. Lung imaging was discussed in a lung MDT with a consultant radiologist, consultant chest physician and consultant general physician. The MDT findings were concordant with an independent over-read by a consultant specialist in cardiothoracic imaging. Clinically significant lung involvement on imaging was defined by MDT on the basis of findings of bilateral fibrosis, bilateral ongoing ground-glass changes or pulmonary emboli. Clinically significant cardiac involvement on CMR was defined as more than borderline left ventricular (LV) systolic dysfunction (LV ejection fraction (LVEF) <50%), ongoing tissue oedema, myocardial infarct on late gadolinium imaging or regional wall motion abnormality.

### Statistical analysis

Statistical analysis was performed using GraphPad Prism (V.8; GraphPad Software, La Jolla, California, USA). Comparison of distinct clinical groups was conducted using Mann-Whitney (non-parametric) or unpaired t-test (parametric) tests. Analysis of contingency tables for diagnostic reliability of CPET in excluding significant lung or cardiac disease was performed by Fisher’s exact test. All data are reported either by median with IQR or by mean±SD. A p value of 0.05 (two-tailed) was taken to indicate statistical significance. Details of the receiver operating characteristic (ROC) analysis of CPET parameters to establish optimal diagnostic criteria are described in the supplementary methods.

## Results

One hundred and thirteen consecutive patients were assessed. The median age was 40 years (IQR 33–47, minimum 19 to maximum 56). Fifty-seven patients (50.4%) had laboratory-confirmed infection (either PCR or antibody testing); a further 14 patients (12.4%) had inpatient radiographic or blood tests typical of COVID-19 infection. Those without a positive PCR belonged to a subgroup with acute illness in March to April 2020 when PCR testing was not available. All had an acute respiratory illness clinically judged to be COVID-19 pneumonitis. Twenty-one patients (19%) were admitted to the hospital for care including supplemental oxygen. Five of these inpatients (4%) were admitted to intensive care unit and four were intubated and ventilated (3.5%). All seven of the group ultimately adjudicated as having the clinically significant disease were admitted to the hospital and three of thm were intubated and ventilated.

The median duration from acute illness onset to assessment was 29 (22–35) weeks. Every patient completed a CPET, spirometry and echocardiogram. Sixty-seven patients had CT chest and dual-energy CTPA. Sixty-seven patients had a CMR scan.

Table 1 presents the patient demographic data, key selected parameters from CPET and 6-min walk distance for the whole patient cohort, patients with clinically significant disease and those with no clinically significant disease. Patients with clinically significant disease were older, by a median of 10 years, but did not differ in other baseline characteristics. About 4% of patients were current smokers; 9% had a diagnosis of asthma; 8% had hypertension; 3% had impaired glucose tolerance and 2% had type 2 diabetes. No patients had a history of coronary artery disease or heart failure.

**Table 1:** Demographic and cardiopulmonary characteristics

	All	Significant cardiopulmonary pathology	No significant cardiopulmonary pathology	Significance (pathology vs no pathology)
No of patients	113	7	106	–
Age (years)	40 (33–47)	50 (45–54)	39 (31–46)	*
Male (%)	95 (84.3)	7 (100)	88 (83.0)	ns
BMI mean	29 (±4.2)	31 (±3.9)	29 (±4.3)	ns
BMI >30 (%)	40 (35)	4 (57)	40 (38)	ns
BMI >35 (%)	12 (9)	0 (–)	12 (11)	ns
No (%) of +ve PCR/AB test	57 (50.4)	5 (71)	52 (49)	ns
Weeks from acute illness to assessment	29 (23–34)	22 (21–25)	29 (23–35.5)	ns
RER	1.17 (±0.06)	1.20 (±0.05)	1.17 (±0.06)	ns
Peak lactate (mmol/L)	12.4 (±2.8)	9.4 (±1.8)	12.7 (±2.7)	ns
V̇O_2_ peak (mL/kg/min)	34.1 (±7.2)	26.7 (±5.1)	34.6 (±7.0)	**
V̇O_2_ % peak predicted	112 (±20)	95 (±15)	113% (±20)	*
Peak workload (W)	244 (±55)	200 (±36)	247 (±55)	*
Peak workload % predicted	101 (±22)	82 (±16)	102 (±22)	*
V̇O_2_ at AT (mL/kg/min)	13.8 (±2.9)	11.8 (±1.8)	14.0 (±2.9)	**
V̇E/V̇CO_2_ slope	28.2 (±5.9)	37.0 (±8.0)	27.6 (±5.3)	***
V̇E/V̇CO_2_ at AT	27.0 (±3.7)	32.8 (±4.1)	26.6 (±3.35)	***
% Breathing reserve†	15 (±13)	7 (±18)	17% (±17)	ns
Alveolar–arterial gradient rest (kPa)	2.5 (±2.0)	3.8 (±2.0)	2.4 (±2.0)	ns
Alveolar–arterial gradient stress (kPa)	2.5 (±2.0)	4.6 (±1.5)	2.3 (±2.0)	**
Resting heart rate (bpm)	85 (±14)	82 (±8.6)	85 (±14)	ns
Peak heart rate (bpm)	173 (±16)	156 (±19)	174 (±15)	**
Peak heart rate % predicted	108 (±9)	103 (±13)	108 (±9)	ns
Heart rate recovery (bpm fall in 60 s)	27 (±13)	28 (±12)	27 (±14)	ns
V̇O_2_/HR% peak predicted	104 (±22)	92 (±7.4)	105 (±22)	ns
Six-min walk test distance (m)	614 (±110)	621 (±122)	613 (±110)	ns

Mean values are presented (±SD); median values are presented with IQR (first to third quartile). Significant differences were tested between seven patients with significant disease and 106 patients with no significant disease. Level of significance for group comparison: ns; *p<0.05; **p<0.01; ***p<0.001.

*p<0.05; **p<0.01; ***p<0.001.

†Breathing reserve: predicted peak minute ventilation−actual peak minute ventilation (L/min).

AT, anaerobic threshold; BMI, body mass index; bpm, beats per minute; HR, heart rate; ns, not significant; RER, respiratory exchange ratio (>1.10 implies excellent maximal effort); VCO_2_, carbon dioxide elimination; VE, minute ventilation (L/min); V̇O_2_, oxygen uptake.

### Pulmonary investigations

Just 7/113 (6%) patients were adjudicated to have clinically significant disease. In six cases this included ongoing bilateral ground-glass changes and five of these had bilateral fibrosis. The patient who had bilateral ground-glass changes and no fibrosis had CT changes in keeping with acute COVID-19 pneumonitis identified on CT imaging 2 days following his CPET (in spite of a negative SARS-CoV-2 nasopharyngeal swab within the previous 5 days). This patient also had subsegmental perfusion deficits in the left lower lobe suggestive of microemboli. The seventh case with significant lung pathology did not have findings typical of COVID-19 pneumonitis. This patient (with a history of childhood asthma which had remained quiescent from age 14, with no medication) had widespread airway inflammation with air-trapping and ‘tree-in-bud’ nodularity of the left lower lobe. This was felt most likely to have resulted from infective (likely bacterial) bronchiolitis (either primary or secondary to COVID-19) leading to the reactivation of airway diseases.

Nineteen (17%) patients had abnormal spirometry (forced vital capacity (FVC) <80% predicted or FEV_1_ (forced expiratory volume in 1 s)/FVC ratio <0.70). Just two of the seven patients with clinically significant lung disease had abnormal spirometry. There was no significant association between spirometry and organ pathology following complete assessment (p=0.33).

### Cardiac investigations

In 113 consecutive transthoracic echocardiograms, there was no severe cardiac pathology. One echocardiogram demonstrated mild aortic regurgitation (trileaflet valve) with normal aortic and LV dimensions and systolic function. Echocardiography, in one patient recovering from severe COVID-19 pneumonitis requiring intubation and ventilation, showed mild elevation of estimated pulmonary artery systolic pressure (right ventricular (RV) systolic pressure 31 mm Hg+right atrial pressure 5–10 mm Hg), with no RV dilatation or dysfunction. Three echocardiograms revealed borderline LV systolic impairment and LV cavity size at the upper limit of normal. In all cases, this occurred in patients with supranormal exercise capacity and high-volume endurance exercise history and following CMR were adjudicated to represent exercise adaptation. Another patient was reported to have borderline RV long-axis systolic function. In this individual, RV size and function were normal on CMR. There were 67 CMR scans, which demonstrated no infarction, no significant systolic impairment (LVEF<50%) or ongoing inflammation. There were four cases (6%) of small-volume late gadolinium enhancement, which were felt to be consistent with previous LV myocarditis. None of these were associated with impaired LV systolic function or regional wall motion abnormality. Furthermore, none of these individuals had pathological ECG changes at the time of clinic assessment.

### Cardiopulmonary exercise tests

There was no difference in the satisfactory completion of maximal exercise tests between clinically significant disease and non-disease groups (RER 1.20 (±0.05) vs 1.17 (±0.06); p=0.59). There was a significant reduction in the absolute, bodyweight-adjusted and %predicted, peak V̇O_2_) (mL/kg/min), and workload achieved associated with the finding of significant disease. Peak V̇O_2_ was 23% lower (26.7 (±5.1) vs 34.6 (±7.0) mL/kg/min, p=0.002; 95% (±15) vs 113% (±20) % of peak predicted, p=0.020). Peak workload was 19% lower (200 (±36) vs 247 (±55) W, p=0.026; 82% (±16) vs 102% (±22) %predicted peak workload, p=0.012).

An even clearer association exists between markers of ventilatory efficiency and the finding of significant disease. The slope of ventilation versus CO_2_ elimination and the value of VE/VCO_2_ at anaerobic threshold (AT) differed between the groups with versus without significant disease by 34% and 23%, respectively (slope 37.0 (±8.0) vs 27.6 (±5.3); p<0.001; VE/VCO_2_ at AT 32.8 (±4.1) vs 26.6 (±3.35); p<0.001). By contrast, the only difference in heart rate, heart rate recovery and O_2_ pulse (V̇O_2_/heart rate) was a lower peak heart rate in the group with significant disease (156 (±19) vs 174 (±15) W; p=0.006), and this difference is largely accounted for by the higher age of the affected group, given that % predicted peak heart rate did not differ (103% (±13) vs 108% (±9); p=0.28).

There was no difference in the 6-min walk distance between the clinically significant disease group and the group with no significant disease identified (621 (±122) vs 613 (±110) m; p=0.86).

**Figure 1:**
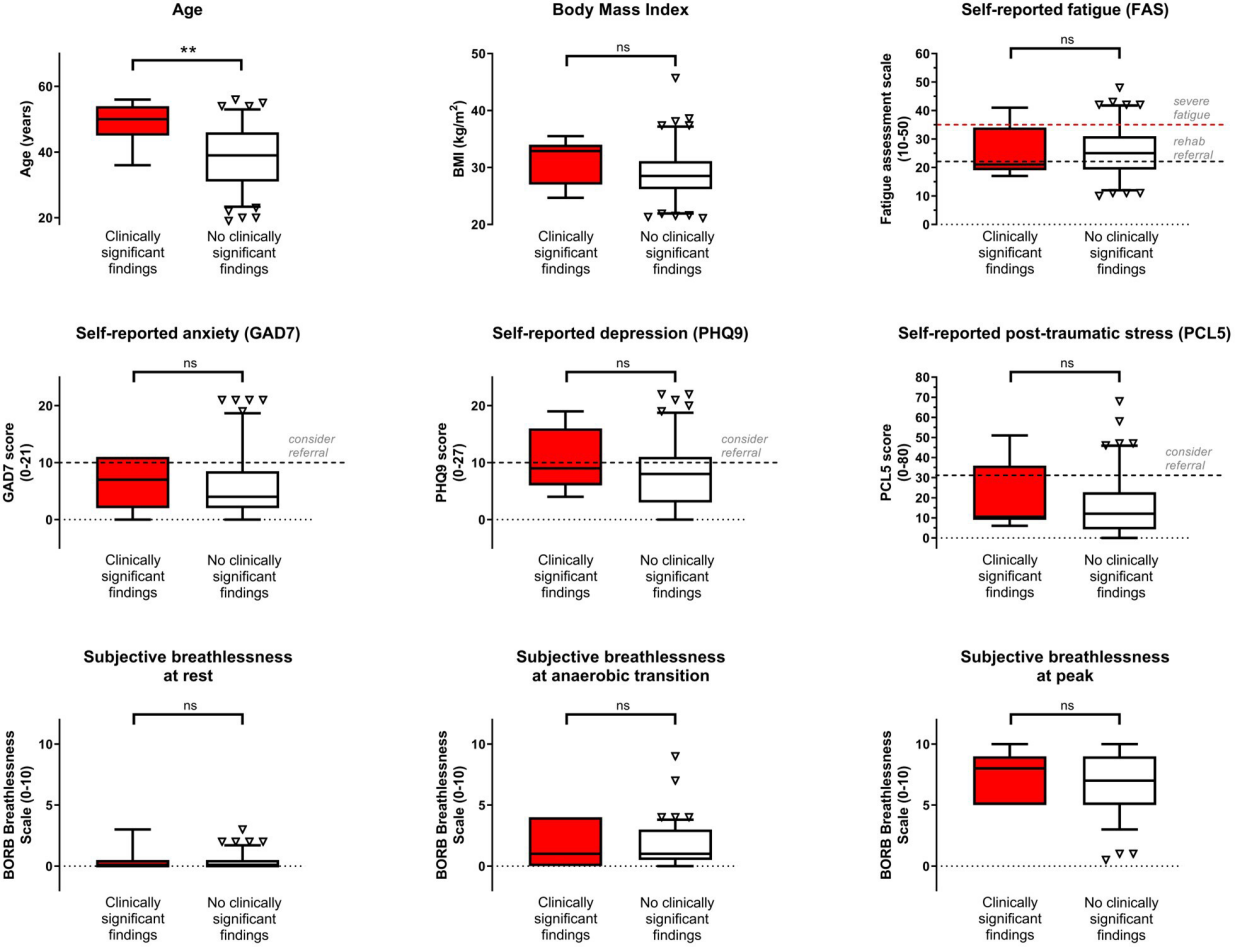
Demographics, symptoms and patient reported outcome measures grouped by presence/absence of clinically significant findings

**Figure 2:**
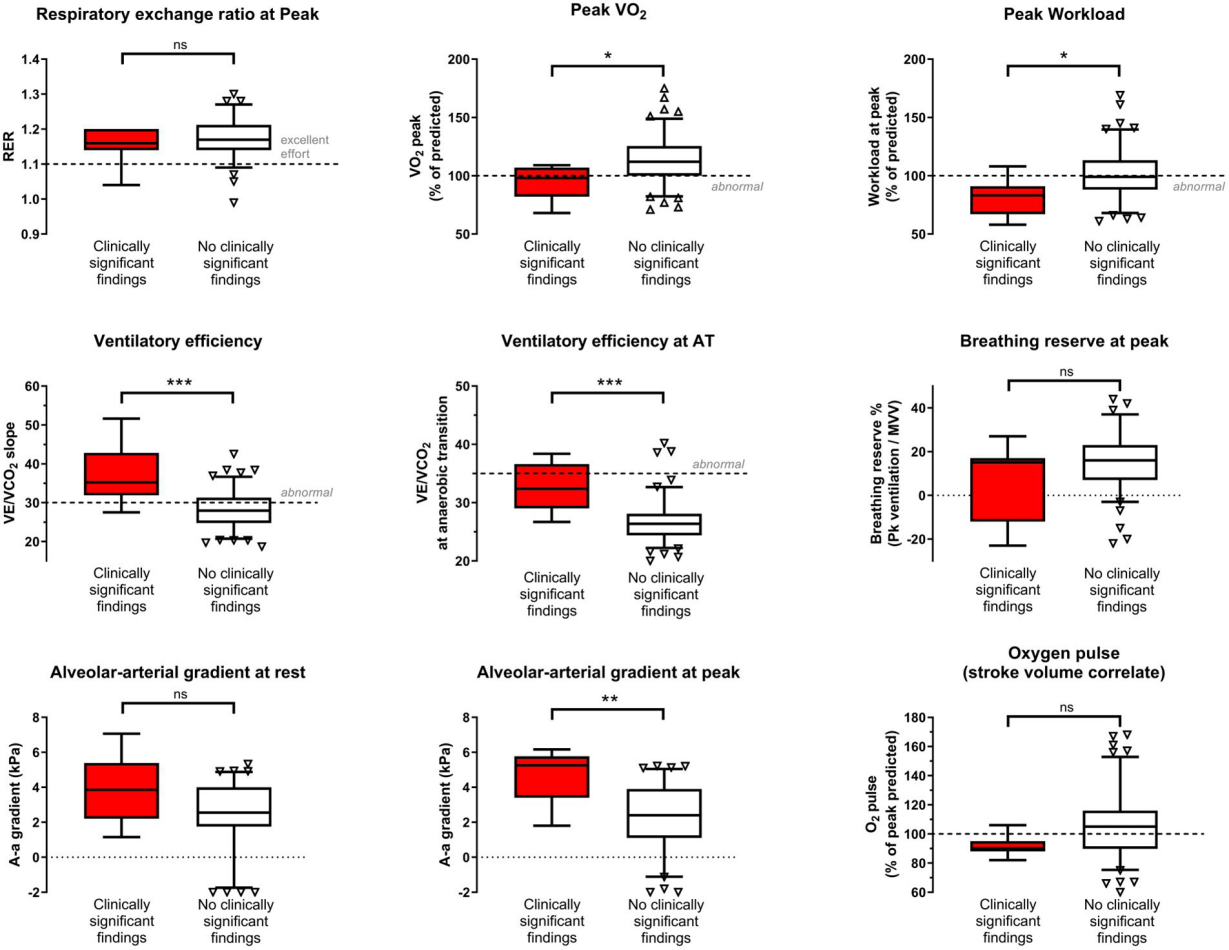
Key cardiopulmonary exercise test findings grouped by presence/absence of clinically significant findings

Figure 1 demonstrates that, except for age, the group in whom significant clinical disease was identified did not differ by BMI, subjective scores of fatigue, anxiety, depression, post-traumatic stress or breathlessness at rest or during maximal exercise. Conversely, Figure 2 demonstrates that clinically significant disease was associated with a significant reduction in peak work and peak oxygen uptake, as well as reduced ventilatory efficiency. While the calculated alveolar–arterial (A-a) gradient of oxygen transport did not differ at rest, at peak there was a 2.3 kPa difference (4.6 (±1.5) kPa vs 2.3 (±2.0) kPa; p=0.002). Breathing reserve at peak (reflective of mechanical ventilatory limitation) did not differ.

**Figure 3:**
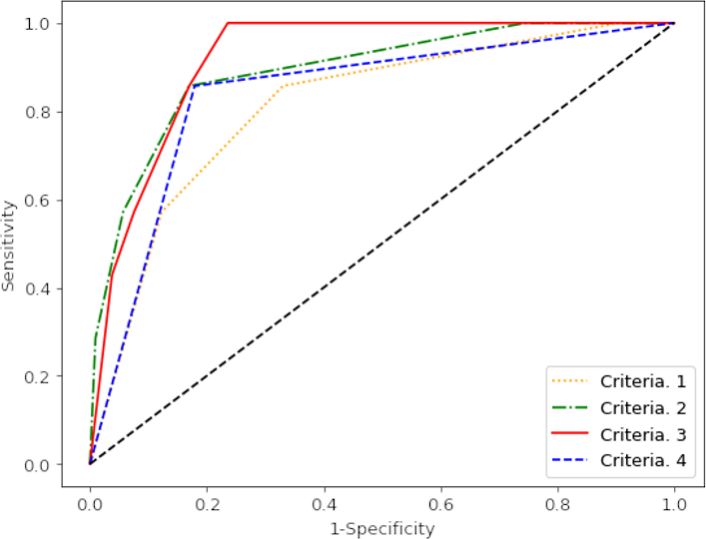
Receiver operating characteristics (ROC) curve for the performance of different CPET based criteria in predicting clinically significant disease

ROC analysis of CPET parameters as predictors of clinically significant disease identified four sets of ‘diagnostic criteria’ that are of clinical interest. The diagnostic performance of these criteria is displayed in Table 2, with their respective ROC curves shown in Figure 3.

**Table 2:** Diagnostic potential of cardiopulmonary exercise testing in the exclusion of clinically significant lung and heart pathology

Criteria	Accuracy	Sensitivity	Specificity	AUC	NPV
Slope >30 and peak predicted V̇O_2_<100%	86%	57%	88%	0.81	97%
OR slope >35 (slope >30 and peak predicted V̇O_2_<100%)	83%	86%	83%	0.89	99%
OR slope >35 OR A-a gradient >4.4 kPa (slope >30 AND peak predicted V̇O_2_ <100%)	78%	100%	78%	0.92	100%
Work predicted <85%	82%	86%	82%	0.84	99%

Accuracy refers to the closeness of the CPET criteria to the frequency of normal or pathological findings on comprehensive clinical imaging. ‘Slope’ refers to the gradient of the function: ventilation (L/min) versus CO_2_ elimination (L/min)—a sensitive measure of ventilatory efficiency. A higher value indicates lower ventilatory efficiency. A-a gradient refers to the estimated gradient of oxygen between the alveoli (PAO_2_) and the arterial blood (PaO_2_) at the point of peak exertion. This is estimated using capillary blood testing from the earlobe. Level of significance for Fisher’s exact test of contingency tables **p<0.01; ***p<0.001.

AUC, area under the receiver operating characteristic curve; CPET, cardiopulmonary exercise testing; NPV, negative predictive value; VO_2_, oxygen uptake.

**Figure 4:**
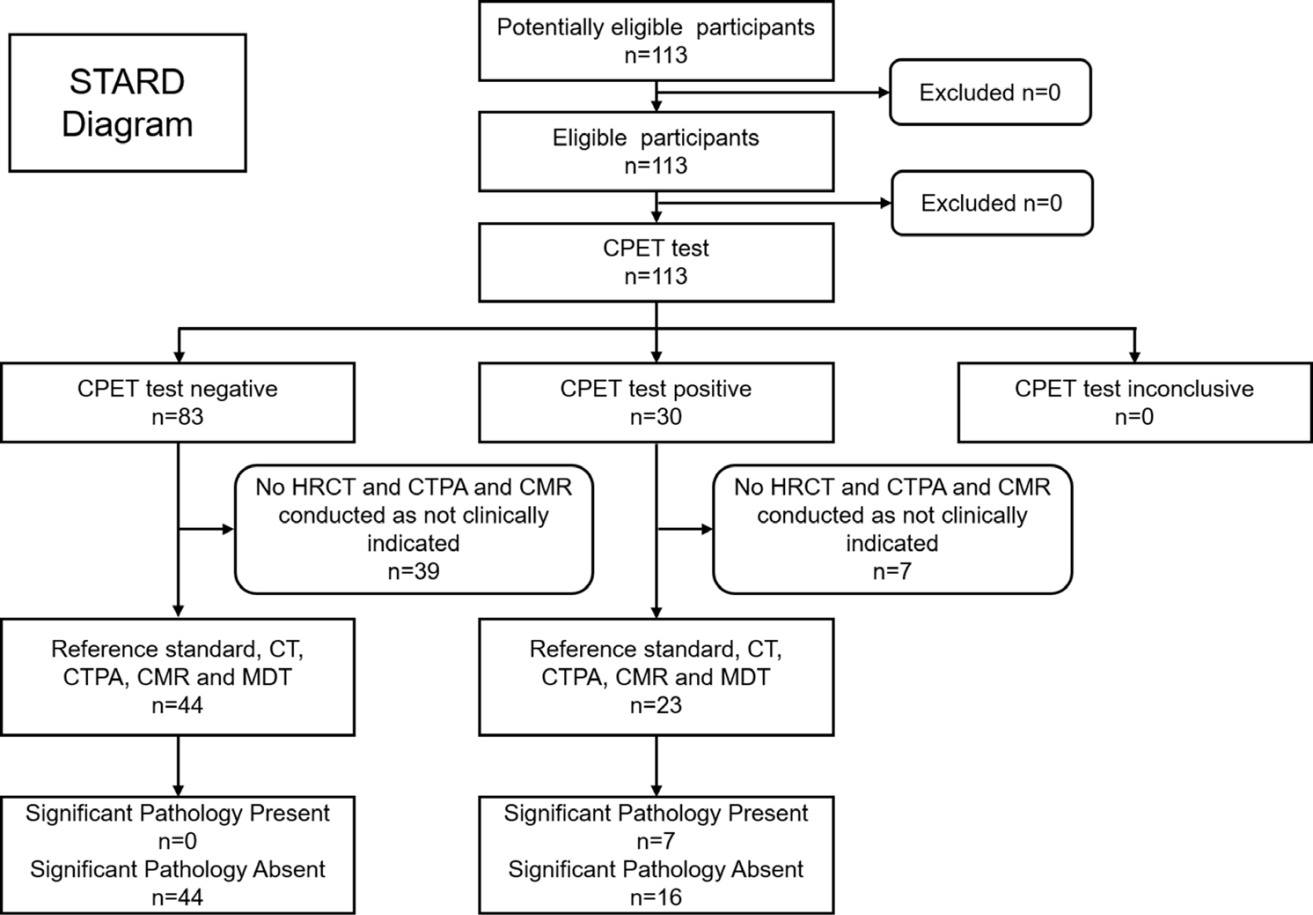


The most sensitive criteria identified patients who were either found to have a V̇O_2_ peak predicted <100% *and* a VE/V̇CO_2_ slope >30.0 *or* either a VE/V̇CO_2_ slope >35.0 in isolation *or* an A-a gradient >4.40 kPa in isolation. The retrospective application of these criteria on the consecutively assessed cohort of 113 patients is detailed in the STARD (Standards for Reporting Diagnostic Accuracy Study) diagram in Figure 4.

## Discussion

In a consecutive cohort of 113 personnel recovering from COVID-19, only seven (6%) had clinically significant heart or lung disease.

Neither symptoms, nor spirometry, nor physician assessment alone were effective in discriminating significant lung disease identified by cross-sectional imaging. By contrast, simple, objective criteria on CPET provided good sensitivity for the prediction of significant lung pathology. Using CPET alone (without any blood gas measurement), sensitivity and specificity of 86% and 83%, respectively, were achieved. Diagnostic accuracy was improved by the addition of an estimate of A-a gradient for oxygen using capillary blood gas testing at peak exercise: sensitivity was 100%, with specificity of 78%. Contrastingly, the self-paced 6-min walk test did *not* differ between the groups with and without significant disease.

Basing assessment of military personnel with prolonged post-COVID symptoms on a cheap, accessible functional test is practical and occupationally appropriate. Such ‘functional triage’ is suitable to guide the requirement/or not for physician-led and/or rehabilitation-led follow-up. Many post-COVID syndrome services are under great pressure and the importance of targeting appropriate scarce resources to those in greatest need should not be underestimated. Recent studies which have also described an abnormal ventilatory efficiency post-COVID have attributed this wholly to altered peripheral chemosensitivity to arterial CO_2_ rather than to cardiopulmonary pathology.^21^ While there is evidence of impaired peripheral oxidative metabolism, this can neither explain the elevated A-a gradient nor impaired gas transfer that has been seen following COVID-19 pneumonitis. The current study used a pragmatic, real-world approach to diagnosing significant clinical pathology and employed an ROC analysis to test the diagnostic potential of CPET parameters to predict these clinically significant findings.

It is important to recognise that this is a retrospective analysis of a modest-sized cohort of patients post-COVID-19. There is some heterogeneity of clinical disease course in this group which is biased toward the male sex (84%), reflecting an Armed Forces population. In common with the majority of clinical pathways assessing post-COVID disease from late February 2020 onwards, only 63% of the group had either SARS-CoV-2 confirmed by laboratory testing (54%) or typical CT/laboratory blood test findings (an additional 9%—such as typical bilateral changes on acute chest CT and lymphopaenia). The fact that only a small proportion of this cohort was ultimately found to have clinically significant disease is an interesting finding in itself. The small number of cases of pathology means that the current findings should be regarded as *hypothesis generating* and they clearly require external validation. Nevertheless, this is a well-characterised, consecutive sample of military patients of working age, in full-time employment all of whom underwent the same, standardised, comprehensive clinical pathway.

CPET provides an important objective measurement of functional capacity and characterisation of cardiopulmonary pathophysiology. Even without recourse to CPET or capillary blood gas analysis, and with access limited to an accurately calibrated bike, which can deliver a ‘ramp’ workload, the attainment of ≥85% predicted workload excludes significant disease with sensitivity and specificity of 86% and 82%. This is an important additional finding because it broadens considerably the number of healthcare settings in which functionally guided triage might be simply and safely undertaken. This approach promises hard-pressed clinicians, dealing with the growing numbers of patients with post-COVID-19 syndrome, a safe, objective tool to assess patients with post-COVID-19 syndrome.

## Data Availability

Data are available on reasonable request. Data may be obtained on approach to the corresponding author, subject to any limitations required on the basis of the group representing a Defence population.
